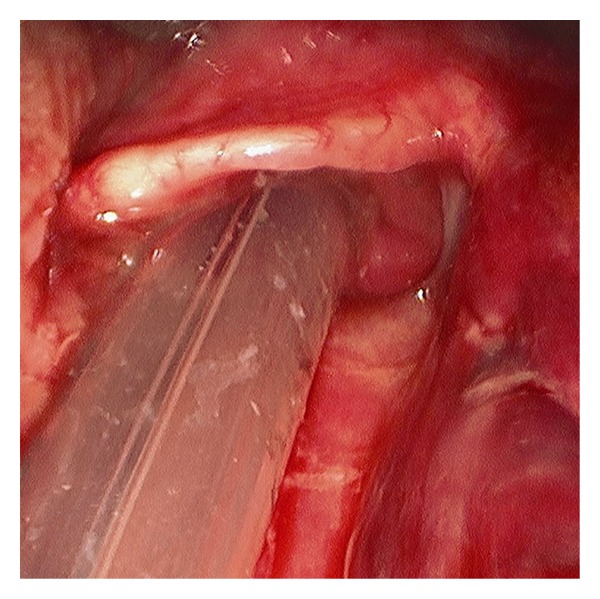# A Rare Complication of Tracheal Intubation: Tongue Perforation

**DOI:** 10.1155/2012/281791

**Published:** 2012-09-29

**Authors:** Loreto Lollo, Tanya K. Meyer, Andreas Grabinsky

**Affiliations:** ^1^Department of Anesthesiology & Pain Medicine, Harborview Medical Center, University of Washington, No. 359724, 325 Ninth Avenue, Seattle, WA 98104, USA; ^2^Department of Otolaryngology, Harborview Medical Center, University of Washington, 325 Ninth Avenue, Seattle, WA 98104, USA

## Abstract

*Aim*. To describe the subsequent treatment of airway trauma sustained during laryngoscopy and endotracheal intubation. *Methods*. A rare injury occurring during laryngoscopy and endotracheal intubation that resulted in perforation of the tongue by an endotracheal tube and the subsequent management of this unusual complication are discussed. A 65-year-old female with intraparenchymal brain hemorrhage with rapidly progressive neurologic deterioration had the airway secured prior to arrival at the referral institution. The endotracheal tube (ETT) was noted to have pierced through the base of the tongue and entered the trachea, and the patient underwent operative laryngoscopy to inspect the injury and the ETT was replaced by tracheostomy. *Results*. Laryngoscopy demonstrated the ETT to perforate the base of the tongue. The airway was secured with tracheostomy and the ETT was removed. *Conclusions*. A wide variety of complications resulting from direct and video-assisted laryngoscopy and tracheal intubation have been reported. Direct perforation of the tongue with an ETT and ability to ventilate and oxygenate subsequently is a rare injury.

## 1. Introduction 

A wide variety of complications resulting from direct and video-assisted laryngoscopy and tracheal intubation have been reported [1, 2]. Although infrequent these injuries include lacerations of the pharyngeal structures such as the soft palate and palatoglossal fold, as well as the esophagus, which can result in severe hemorrhage that obscures visualization during laryngoscopy for tracheal intubation [3, 4]. These injuries can create a false lumen with potential for catastrophic outcomes such as pneumomediastinum, infection, or even lack of ventilation [5, 6]. Other infrequent injuries have resulted in hoarseness or areas of oropharyngeal numbness attributed to unilateral and bilateral palsies of the recurrent laryngeal and lingual nerves [7, 8]. Arytenoid dislocation and fractures of the thyroid cartilage and tracheal rings have also been described [9–11]. A rare injury occurring during laryngoscopy and endotracheal intubation that resulted in perforation of the tongue by an endotracheal tube, the subsequent management of this unusual complication, is reported.

## 2. Case Description

A 65-year-old Caucasian female with ASA physical status 2 sustained an intraparenchymal brain hemorrhage with rapidly progressive neurologic deterioration that required the airway to be secured. Tracheal intubation was performed prior to arrival at the admitting referral institution. A chart review of placement of the endotracheal tube (ETT) did not document any difficulty or injury with the procedure.

A chart review after admission documented preanesthetic airway assessment for unrelated surgery seven years prior. She was classified as a Mallampati class 2 airway. Tracheal intubation at that time was hindered by small mouth opening after induction, an anteriorly positioned glottic opening with a grade 2 laryngoscopic view by the Cormack-Lehane criteria, and inability to place an ETT through the glottis without the assistance of a gum elastic bougie.

**Figure 1:**
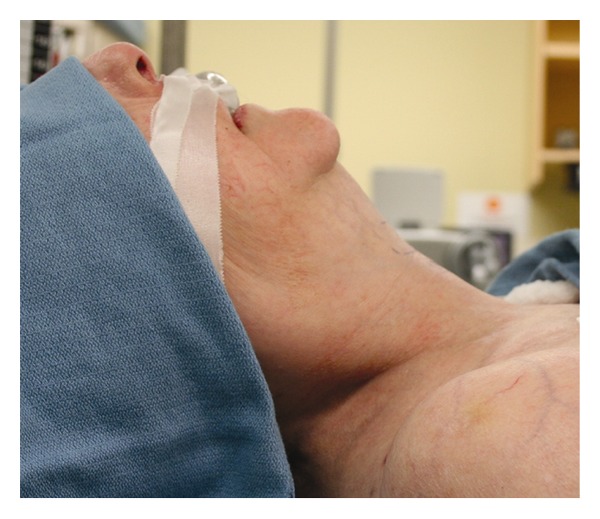


**Figure 2:**
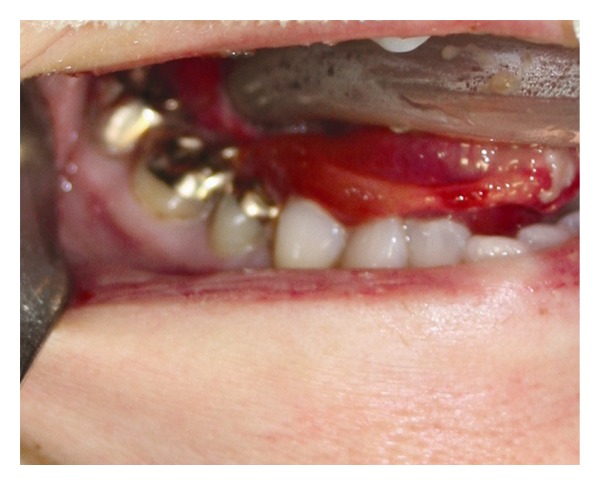


**Figure 3:**
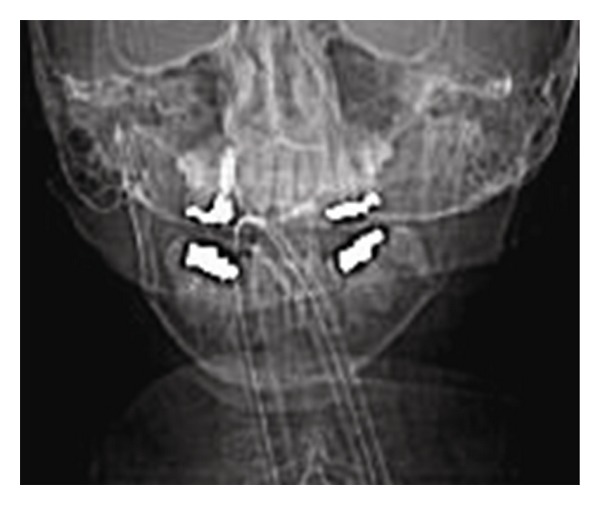


During the current admission to the intensive care unit, it was noted that the ETT pierced the patient's tongue. Anesthesiology and otolaryngology services were consulted for ETT exchange and evaluation of the tongue injury. Inspection of the oral cavity was restricted by limited mouth opening, a short thyromental distance, and crowded dentition (Figure 1). A 7.0 mm internal diameter ETT on the right side of the oral cavity pierced through the body of the tongue with a 4 mm pedicle of tissue bridging around the ETT. Ecchymosis and edema of the surrounding oropharyngeal tissue made further evaluation difficult but the ETT was in correct position in the trachea (Figure 2). Anteroposterior radiographs of the facial structures demonstrated the ETT to be deviated to the right side of the mandible instead of that in the midline position that the ETT bite block maintains for patients mechanically ventilated with an ETT (Figure 3). Oxygenation and ventilation remained normal at all times during the hospital stay. 

**Figure 4:**
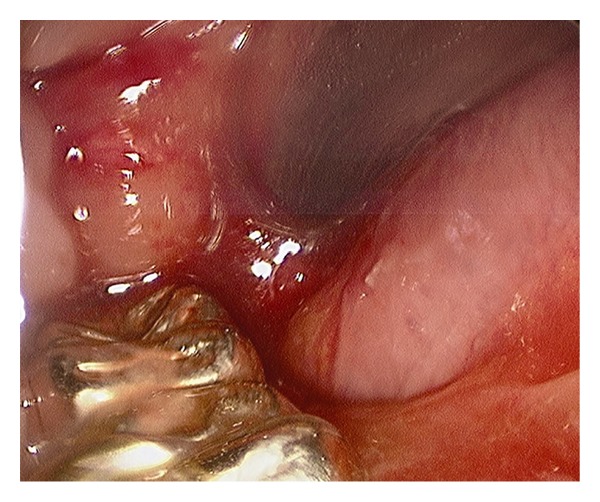


The patient's brain injury had resulted in loss of the gag reflex but the cough reflex remained intact. Consent to perform elective tracheostomy followed by operative direct laryngoscopy (DL), removal of the ETT, and repair of injury to the oropharyngeal structures was obtained, and the patient was transferred to the operating room on hospital day 2. DL demonstrated the ETT traversing the floor of the mouth through a laceration at the junction of the floor of the mouth and the posterior tongue on the right side. The ETT appropriately entered the laryngeal opening (Figure 4). Both the Lindholm and anterior commissure laryngoscopes allowed visualization of the epiglottis, but tilting of the epiglottis to expose the laryngeal inlet or visualization of the laryngeal opening was not possible (Figure 5).

The patient had normal tracheal anatomy, and a standard tracheotomy with placement of a number 4 cuffed Shiley tracheostomy tube was performed. After securing the tracheostomy tube, the ETT was removed and repeat DL revealed no additional injuries. There was no significant bleeding from the tongue or floor of the mouth. The patient had scant drainage of serosanguinous secretions from the laceration site that resolved spontaneously. The patient was placed on palliative care due to the severity of the brain injury and expired on the fifth hospital day. 

## 3. Discussion 

Dental injury is the most common complication of DL for tracheal intubation and can result in aesthetic and functional sequelae. Rarely, aspiration of dental fragments can result in pulmonary complications such as pneumonia and atelectasis. Injuries to cartilaginous and neurologic structures of the laryngopharynx during DL and ETT placement are rare but associated with more serious outcomes due to their importance for phonation and airway protection.

Mucosal injuries due to shearing or traction forces applied by the laryngoscope can cause laceration of oropharyngeal tissue. Penetrating injuries can be caused by the ETT, with or without a stylet introducer. Hemorrhage ensuing from an oropharyngeal mucosal laceration may obscure the visual field during laryngoscopy and result in the creation of a false lumen. Inability to ventilate and subcutaneous and mediastinal emphysema may occur if ventilation is attempted with an ETT placed within this false lumen. Delayed sequelae include pain and extension of infection to adjacent structures such as the submental space and mediastinum. 

Because this individual had a secure airway with adequate oxygenation and ventilation, the surgical team decided to perform subsequent manipulations electively in the operating room. This would have allowed for the best possible conditions to control hemorrhage and repair mucosal damage. The presence of the ETT possibly maintained tamponade of hemorrhage from the tongue perforation. Adequate hemostasis was present at the time of removal of the ETT and further operative repair of the tongue laceration was not deemed necessary. Postoperative monitoring of the patient was continued in order to observe any signs of infection resulting from tongue perforation.

## Figures and Tables

**Figure 5:**